# Retrospective Multicenter Analysis of Withdrawal Syndrome in Parkinson’s Disease Patients After Cessation of Deep Brain Stimulation

**DOI:** 10.3390/diagnostics16040644

**Published:** 2026-02-23

**Authors:** Hatice Ömercikoğlu Özden, Fatma Nazlı Durmaz Çelik, Fatma Şeyda Üstüner, Galip Yardımcı, Orhan Abdullah Omar Tbh Bash, Serhat Özkan, Murat Vural, Fatih Bayraklı, Dilek Günal

**Affiliations:** 1 Department of Neurology, School of Medicine, Marmara University, Basibuyuk Mah. Maltepe Basıbüyük Yolu Sok. No:9/2 Maltepe, 34854 Istanbul, Turkey; fatma.seyda@hotmail.com (F.Ş.Ü.); galipyardimci@hotmail.com (G.Y.); incegunal@yahoo.com (D.G.); 2 Department of Neurology, School of Medicine, Eskisehir Osmangazi University, 26040 Eskisehir, Turkey; doktornazli@hotmail.com (F.N.D.Ç.); timberwolf566@yahoo.com (O.A.O.T.B.); scozkan@gmail.com (S.Ö.); 3 Department of Neurosurgery, School of Medicine, Eskisehir Osmangazi University, 26040 Eskisehir, Turkey; muratvural888@gmail.com; 4 Department of Neurosurgery, School of Medicine, Marmara University, 34854 Istanbul, Turkey; fbayrakli@gmail.com

**Keywords:** Parkinson’s disease, deep brain stimulation, withdrawal syndrome

## Abstract

**Background:** Abrupt cessation of deep brain stimulation (DBS) in Parkinson’s disease (PD), most commonly due to implantable pulse generator (IPG) battery depletion, may lead to DBS withdrawal syndrome (DBS-WDS). However, withdrawal syndrome does not occur in all patients following stimulation cessation. **Methods:** We retrospectively analyzed 210 PD patients treated with DBS. Patients with documented stimulation cessation were evaluated for the presence of withdrawal syndrome based on established clinical criteria. Demographic, disease-related, and treatment characteristics were assessed, and descriptive analysis was conducted on severe cases requiring intensive care. **Results:** DBS battery shutdown occurred in 28 patients (13.3%). Most patients did not develop withdrawal syndrome and experienced only transient motor worsening. Severe DBS-WDS requiring intensive care was rare, occurring in only three patients (1.4%). Battery shutdown alone did not predict withdrawal, nor was preoperative levodopa equivalent daily dose associated with withdrawal risk. **Conclusions:** DBS battery shutdown is usually not accompanied by withdrawal syndrome, and severe DBS-WDS is uncommon. Proactive battery management may help to prevent this rare but serious complication.

## 1. Introduction

Parkinson’s disease (PD) is a heterogeneous and multifactorial neurodegenerative disorder characterized not only by dopaminergic neuronal loss in the substantia nigra pars compacta but also by widespread extranigral pathology. Beyond the classical dopaminergic hypothesis, several pathophysiological mechanisms have been proposed, including neuroinflammation, mitochondrial dysfunction, oxidative stress, impaired proteostasis, and metabolic dysregulation. Emerging evidence suggests that glycemic variability and recurrent hypoglycemic episodes may contribute to neurodegeneration through oxidative stress pathways and inflammatory cascades, potentially influencing the clinical expression of parkinsonian syndromes [1]. These mechanisms may differentially affect disease progression and treatment responsiveness across PD subtypes.

Deep brain stimulation (DBS) of the subthalamic nucleus (STN) or globus pallidus internus (GPi) is an established and effective therapy for advanced Parkinson’s disease (PD), leading to sustained improvement in motor symptoms and a reduction in dopaminergic medication requirements [2,3,4]. With an increasing number of patients treated with long-term DBS, attention has shifted toward complications related to hardware malfunction or abrupt cessation of stimulation.

Although DBS is generally considered a reversible and adjustable treatment, sudden interruption of chronic DBS—most commonly due to implantable pulse generator (IPG) battery depletion, hardware malfunction, or explantation for infection—may precipitate a rare but potentially life-threatening condition known as DBS withdrawal syndrome (DBS-WDS) or malignant DBS withdrawal syndrome [5,6,7,8,9]. Clinically, DBS-WDS is characterized by acute and profound worsening of parkinsonism, including severe akinesia and rigidity, dysphagia, altered mental status, autonomic instability, hyperthermia, and elevated creatine kinase levels, frequently mimicking neuroleptic malignant syndrome or parkinsonism–hyperpyrexia syndrome [5,7,10].

Several case reports and small case series have demonstrated that DBS-WDS is often refractory to aggressive dopaminergic therapy, including high-dose levodopa or continuous apomorphine infusion, whereas rapid restoration of neurostimulation through IPG replacement or reimplantation leads to dramatic clinical improvement [6,8,11,12]. Delayed restoration of stimulation has been associated with severe complications and fatal outcomes in some cases [7,9].

Importantly, not all patients experience DBS-WDS following stimulation cessation. While abrupt DBS interruption invariably causes motor worsening, only a subset of patients develop a malignant withdrawal syndrome [13]. The largest comparative analysis to date suggested that longer Parkinson’s disease duration, prolonged DBS exposure, older age, and more advanced motor impairment may increase vulnerability to DBS-WDS [13]. These findings support the concept that DBS-WDS is not solely the result of stimulation interruption, but rather reflects a complex interaction between disease progression, chronic neuromodulation, and individual susceptibility.

The pathophysiological mechanisms underlying DBS-WDS remain incompletely understood. Proposed hypotheses include progressive desensitization of dopaminergic pathways, maladaptive plastic changes within basal ganglia–thalamocortical networks, and increasing functional dependence on electrical stimulation after long-term DBS [7,13,14]. According to this “DBS dependency” model, abrupt withdrawal of stimulation unmasks a critically fragile motor network that cannot be adequately compensated by pharmacological therapy alone.

Despite growing awareness of the risk, systematic data comparing patients who develop DBS-WDS with those who tolerate DBS cessation without withdrawal are limited. Identifying clinical and treatment-related factors that protect against DBS-WDS is essential for improving risk stratification, optimizing battery management strategies, and preventing this rare but devastating complication.

Therefore, the aim of the present study was to compare Parkinson’s disease patients who underwent chronic DBS and developed withdrawal syndrome following IPG depletion with those who experienced stimulation cessation without withdrawal, in order to identify potential protective and predisposing factors.

## 2. Materials and Methods

### 2.1. Study Design and Patient Selection

This retrospective observational study was conducted at tertiary movement disorders centers with established DBS programs. The medical records of patients with Parkinson’s disease who underwent DBS implantation and subsequent follow-up were reviewed.

Patients were eligible for inclusion if they met the following criteria:

1. Diagnosis of idiopathic Parkinson’s disease according to established clinical criteria.
2. Motor severity was assessed using the Unified Parkinson’s Disease Rating Scale Part III (UPDRS-III), and disease stage was determined according to the Hoehn and Yahr (H&Y) classification at the time of DBS implantation and at the occurrence of withdrawal symptoms.
3. Bilateral DBS implantation targeting the STN or GPi.
4. Documented cessation of DBS due to IPG battery depletion, hardware malfunction, or explantation for infection.
5. Availability of detailed clinical documentation during the stimulation-off period.

Patients were excluded if DBS was intentionally switched off for programming or experimental purposes, or if clinical data during the stimulation-off interval were incomplete.

### 2.2. Definition of DBS Withdrawal Syndrome

DBS withdrawal syndrome (DBS-WDS) was defined as an acute clinical deterioration occurring after abrupt cessation of chronic DBS, characterized by the following symptoms:

- Severe worsening of parkinsonism with marked akinesia and rigidity;
- Dysphagia or inability to maintain oral dopaminergic therapy;
- Altered mental status and/or autonomic instability;
- Hyperthermia and/or elevated creatine kinase levels;
- Poor or absent response to intensified dopaminergic treatment.

This definition was based on previously published clinical descriptions of malignant DBS withdrawal and DBS-related parkinsonism–hyperpyrexia syndromes [5,6,7,8,9,13].

Patients who exhibited motor worsening without systemic or life-threatening features were classified as non-withdrawal DBS interruption cases.

### 2.3. Study Groups

Patients were categorized into two groups, as follows:

- **Withdrawal group**—patients who developed DBS-WDS following stimulation cessation.
- **Non-withdrawal group**—patients who experienced DBS interruption without developing DBS-WDS.

### 2.4. Data Collection

The following variables were extracted from medical records:

- Demographic variables—age and sex.
- Disease-related variables—age at PD onset, Hoehn and Yahr (H&Y) stage, disease duration at DBS implantation, and disease duration at IPG depletion.
- DBS-related variables—target (STN vs. GPi), duration of DBS therapy, type of IPG, reason for stimulation cessation, and duration of stimulation interruption.
- Treatment variables—levodopa equivalent daily dose (LEDD) before DBS, after DBS, and during the stimulation-off period.
- Clinical outcomes—presence of hyperthermia, rhabdomyolysis, need for intensive care, timing of IPG replacement or reimplantation, and mortality.

### 2.5. Statistical Analysis

Continuous variables are expressed as mean ± standard deviation or median (interquartile range), as appropriate. Categorical variables are presented as frequencies and percentages. Group comparisons were performed using appropriate parametric or non-parametric tests. A *p*-value < 0.05 was considered statistically significant.

### 2.6. Ethical Considerations

This study was conducted in accordance with the Declaration of Helsinki and approved by the local institutional ethics committees (Protocol code: 09.2025.25-0636). Due to the retrospective nature of the study, informed consent requirements were waived where applicable.

## 3. Results

### 3.1. Study Population and Overall Characteristics

A total of 210 patients with Parkinson’s disease (PD) who underwent deep brain stimulation (DBS) were included in the present analysis. All patients had comprehensive demographic, clinical, and device-related data available for evaluation. The cohort was predominantly male, with a long disease duration and advanced-stage PD at the time of DBS implantation. During follow-up, DBS battery shutdown or depletion was documented in 28 patients (13.3%), whereas 182 patients (86.7%) did not experience battery shutdown. At the time of DBS implantation, the mean UPDRS-III score was 29.2 ± 16.6, and the median Hoehn and Yahr (H&Y) stage was 3 (range of 2–4). At withdrawal onset, motor severity worsened, with the mean UPDRS-III score increasing to 33.1 ± 11.5. Baseline demographic and clinical characteristics of patients who did and did not experience battery shutdown are summarized in Table 1.

Overall, patients who did and did not experience battery shutdown were comparable in terms of age, sex distribution, Parkinson’s disease duration, age at disease onset, and preoperative levodopa equivalent daily dose (Preop LEDD). The lack of significant differences between the two groups for these variables indicated that battery shutdown was not associated with distinct baseline demographic or disease-related features.

### 3.2. Battery Shutdown and Clinical Outcomes

Among the patients with documented battery shutdown, the predominant clinical outcome was the absence of withdrawal syndrome. The majority of these patients remained clinically stable or experienced only transient worsening of motor symptoms following stimulation interruption. Importantly, they did not develop hyperthermia, severe autonomic instability, rhabdomyolysis, or other systemic complications that would necessitate intensive care management. Severe DBS withdrawal syndrome requiring intensive care unit (ICU) admission was rare, occurring in only three patients, which corresponded to 1.4% of the entire cohort and a small minority of patients with documented battery shutdown. Thus, in this cohort, DBS battery shutdown was usually not accompanied by withdrawal syndrome, and malignant withdrawal represented an exceptional clinical course rather than the rule.

### 3.3. Characteristics of Patients with Severe DBS Withdrawal Syndrome

The three patients who developed severe DBS withdrawal syndrome exhibited a rapidly progressive clinical deterioration characterized by profound akinesia and rigidity, marked functional decline, impaired oral intake, and features of systemic or autonomic instability, ultimately requiring ICU-level care. Given the very small number of ICU-requiring withdrawal cases (*n* = 3), the data of these patients were analyzed descriptively, without inferential statistical testing, in accordance with the current accepted methodological standards for rare events. Their individual clinical and device-related characteristics are presented in Table 2. The stimulation parameters prior to battery depletion were as follows: In Case 1, bilateral STN stimulation was delivered with right STN contact 9 (−)/IPG case (+) at 3.7 V, 60 µs, and 130 Hz, and left STN contact 2 (−)/IPG case (+) at 2.9 V, 60 µs, and 130 Hz; device interrogation confirmed that the implantable pulse generator (IPG) had reached end-of-life (EOL) status. In Case 2, stimulation was programmed as right STN contacts 9 (−)/8 (−) at 2.0 V, 60 µs, and 130 Hz, and left STN contacts 2 (−)/1 (−) at 2.1 V, 60 µs, and 130 Hz. In Case 3, bilateral STN stimulation was set at right STN contact 2 (−)/IPG case (+) at 2.7 mA, 60 µs, and 174 Hz, and left STN contact 3 (+)/5 (−) at 2.8 mA, 60 µs, and 174 Hz.

Notably, although all three patients fulfilled the clinical criteria for malignant DBS withdrawal, battery shutdown alone did not reliably predict this outcome, as most patients with battery depletion did not develop withdrawal syndrome. This observation underscores the importance of distinguishing battery shutdown as a technical event from true severe DBS withdrawal syndrome, which appears to represent a distinct and uncommon clinical entity.

### 3.4. Comparison of Patients with and Without Battery Shutdown

A detailed comparison of patients with documented battery shutdown versus those without is shown in Table 1. There were no significant differences between the two groups with respect to the following clinical characteristics:

- Age;
- Sex distribution;
- Parkinson’s disease duration;
- Age at disease onset;
- Preop LED.

Specifically, preoperative dopaminergic burden, as reflected by Preop LED, was similar between the two groups. This finding suggests that baseline medications did not predispose patients to the risk of battery shutdown and were not associated with the benign clinical course observed in most patients after battery shutdown.

### 3.5. Preoperative Dopaminergic Burden and Withdrawal Risk

Preoperative dopaminergic burden was further explored in relation to withdrawal outcomes. Across the entire cohort, Preop LED values showed wide interindividual variability but did not differ significantly between patients with and without documented battery shutdown. Among the three patients who exhibited severe withdrawal syndrome, Preop LED values did not follow a consistent pattern and overlapped with those observed in patients who experienced battery shutdown but did not develop withdrawal syndrome. Given the rarity of severe withdrawal events, no definitive association between Preop LED and ICU-requiring withdrawal could be established in this dataset.

## 4. Discussion

Parkinson’s disease exhibits substantial clinical heterogeneity, and the tremor-dominant (TD), postural instability and gait disorder (PIGD), and akinetic-rigid subtypes demonstrate different progression rates and treatment responses. Advanced PD patients undergoing DBS often belong to more aggressive phenotypes such as the PIGD subtype, which is associated with faster axial deterioration and non-motor burden. Therefore, the risk and clinical presentation of DBS withdrawal syndrome may vary according to the underlying PD subtype, potentially reflecting differences in disease biology and network-level dysfunction [15,16]

The pathophysiology of DBS withdrawal syndrome remains incompletely understood. Abrupt cessation of chronic STN stimulation has been associated with life-threatening akinetic crises and severe parkinsonism refractory to dopaminergic therapy in case reports and small series, suggesting disruption of basal ganglia–thalamocortical network modulation as a key mechanism [6,13]. Additional reports have described similar malignant withdrawal events in individual patients, supporting the notion that sudden withdrawal unmasks a critically fragile motor network in advanced PD [5]. Moreover, chronic DBS is hypothesized to induce adaptive neuroplastic changes and functional dependency on stimulation, and abrupt discontinuation may lead to maladaptive network instability. Metabolic and inflammatory vulnerabilities, such as those associated with glycemic variability and hypoglycemia in PD, have also been implicated in exacerbating clinical severity and disease progression [1]. These mechanisms are further supported by broader clinical observations of acute decompensation following DBS cessation, emphasizing the need for early detection of battery depletion and emergency intervention [17].

In this retrospective analysis of Parkinson’s disease patients treated with deep brain stimulation, we found that abrupt DBS battery shutdown is not commonly associated with withdrawal syndrome, and that severe DBS withdrawal requiring intensive care admission is a rare event, observed in only three patients in our cohort. These findings underscore that DBS withdrawal syndrome represents a distinct and uncommon clinical entity, rather than an inevitable consequence of stimulation cessation.

### 4.1. Battery Shutdown Does Not Inevitably Lead to Withdrawal Syndrome

Although abrupt cessation of chronic DBS invariably results in motor worsening, our data clearly show that the majority of patients who experienced battery shutdown did not develop withdrawal syndrome and remained clinically stable or experienced only transient symptom exacerbation. This observation aligns with previous reports suggesting that only a subset of patients exposed to stimulation interruption develop malignant DBS withdrawal syndrome (DBS-WDS) [7,13]. Importantly, many earlier publications have focused primarily on case reports or small case series of DBS-WDS, which may overrepresent severe outcomes [5,6,7,8,9]. In contrast, our cohort-based analysis provides a broader perspective, demonstrating that benign clinical courses following battery depletion are far more common than life-threatening withdrawal, a finding that has important implications for clinical counseling and risk stratification.

### 4.2. Severe DBS Withdrawal Syndrome as a Rare but Distinct Clinical Entity

In our cohort, only three patients (1.4%) developed severe DBS withdrawal syndrome requiring ICU-level care. These patients exhibited the classical features described in the literature, including rapid and profound worsening of parkinsonism, dysphagia, altered mental status, and systemic or autonomic instability, closely resembling previously reported malignant DBS withdrawal and parkinsonism–hyperpyrexia syndromes [5,7,10]. Given the extremely small number of severe events, we intentionally refrained from conducting inferential statistical analyses for predictors of ICU-requiring withdrawal and instead analyzed these cases descriptively. This approach is methodologically appropriate and avoids overinterpretation of rare outcomes. Notably, battery shutdown alone did not reliably predict severe withdrawal, as most patients with documented battery shutdown did not progress to this life-threatening condition.

### 4.3. Preoperative Dopaminergic Burden and Withdrawal Risk

Preoperative dopaminergic burden, assessed based on levodopa equivalent daily dose (Preop LED), has been hypothesized to influence vulnerability to DBS-WDS by reflecting advanced disease stage or dopaminergic dependency [13]. However, in our cohort, Preop LED did not differ between patients with and without documented battery shutdown, nor did it show a consistent pattern among the three patients with severe withdrawal after battery shutdown. These findings suggest that baseline dopaminergic requirements alone are insufficient to explain susceptibility to DBS-WDS, supporting previous observations that pharmacological escalation is often ineffective once malignant withdrawal has developed [6,8]. Instead, DBS-WDS appears to reflect a complex interaction between chronic neuromodulation, disease progression, and individual network vulnerability.

### 4.4. Pathophysiological Considerations and DBS Dependency

The mechanisms underlying DBS withdrawal syndrome remain incompletely understood. Several authors have proposed that long-term DBS induces maladaptive plastic changes within the basal ganglia–thalamocortical networks, leading to increasing functional dependence on electrical stimulation over time [7,13,14]. According to this “DBS dependency” hypothesis, abrupt stimulation cessation unmasks a critically fragile motor network that cannot be adequately compensated by dopaminergic therapy alone. Our findings are consistent with this conceptual framework. The rarity of severe withdrawal syndrome among patients who experienced battery shutdown suggests that only a subset of patients reaches a state of extreme DBS dependency, whereas most patients retain sufficient compensatory capacity to tolerate stimulation interruption without catastrophic deterioration.

### 4.5. Clinical Implications

The results of this study have several important clinical implications. First, clinicians should recognize that battery shutdown is usually not associated with withdrawal syndrome, which may help to reduce unnecessary alarms when stimulation interruption occurs. Second, the rarity of severe DBS-WDS highlights the importance of rapid identification and urgent intervention in the small subset of patients who do develop malignant withdrawal syndrome, as timely IPG replacement or reimplantation remains the most effective treatment [8,11]. Finally, our findings emphasize the need to distinguish technical events (battery depletion or hardware issues) from true DBS withdrawal syndrome, as conflating these entities may lead to misinterpretation of risk and inappropriate management strategies.

### 4.6. Limitations

This study has several limitations that should be acknowledged. First, the retrospective design limits causal inference and may introduce selection bias. Second, the relatively small sample size reduces statistical power and may limit generalizability to the broader PD population. Third, detailed metabolic parameters and inflammatory biomarkers were not systematically recorded, precluding mechanistic interpretation. Fourth, the absence of a control group who did not experience DBS withdrawal limits comparative analysis. Finally, subtype stratification was insufficiently powered for subgroup analysis, and future prospective multicenter studies are warranted to validate these findings.

## 5. Conclusions

In this cohort, DBS battery shutdown was usually not accompanied by withdrawal syndrome, and severe DBS withdrawal syndrome requiring intensive care was rare. These findings indicate that abrupt stimulation cessation alone is insufficient to precipitate malignant withdrawal. However, when withdrawal does occur, it represents a distinct and potentially life-threatening complication. Our data highlight the importance of proactive battery management, particularly in patients with non-rechargeable IPGs approaching 4–5 years of use. Close battery monitoring, timely replacement, and appropriate patient education may help to prevent unexpected battery depletion and reduce the risk of withdrawal syndrome.

## Figures and Tables

**Table 1:** Baseline characteristics of the study cohort according to the DBS battery shutdown status.

Variable	Battery Shutdown (*n* = 28)	No Battery Shutdown (*n* = 182)
Age, years	62 (55–68.5)	66 (60–72)
Male sex, *n* (%)	17 (60.7%)	118 (64.8%)
Female sex, *n* (%)	11 (39.3%)	64 (35.2%)
Parkinson’s disease duration, years	20.5 (15.8–25.3)	19 (15–22)
Age at PD onset, years	41.5 (36–47)	46 (40–52)
Preoperative LEDD, mg	965 (737.5–1510)	965 (700–1249)
Severe withdrawal requiring ICU, *n* (%)	3 (10.7%)	0 (0%)

**Table 2:** Clinical characteristics of patients with severe DBS withdrawal syndrome requiring ICU admission (*n* = 3).

Case	Age/Sex	PD Duration (years)	DBS Duration (years)	LEDD (preDBS)	Cause of Stimulation Cessation	Clinical Presentation	Outcome
1	73/Female	13	7	775 mg	IPG depletion	Rigidity, bradykinesia, dysphagia, and confusion (10 days)	Improved after urgent IPG replacement
2	31/Male (PARK2)	10	2	1275 mg	IPG infection	Rigidity, tremor, aspiration pneumonia, and respiratory distress (5 days)	Clinical deterioration until re-implantation
3	54/Male	11	4	1250 mg	IPG depletion	Tremor, gait disturbance, and feeding difficulty	Improved after IPG replacement

Abbreviations: DBS, deep brain stimulation; IPG, implantable pulse generator; PD, Parkinson’s disease; LEDD, levodopa equivalent daily dose.

## Data Availability

The datasets generated during and/or analyzed during the current study are available from the corresponding author upon reasonable request.
